# Choline chloride-based deep eutectic solvents for efficient anthocyanin recovery from black rice: process, kinetics and nanoliposomal formulation

**DOI:** 10.1098/rsos.260927

**Published:** 2026-10-14

**Authors:** Quang-Hieu Tran, Ba Thanh Nguyen, Dat Pham Quoc, Nguyen Quynh Dao, Nguyen Thi Ngoc Tram, Nguyen Thanh Danh, Nguyen Quoc Thang, Le Van Tan

**Affiliations:** ^1^ Faculty of Chemical Engineering, Industrial University of Ho Chi Minh City, Ho Chi Minh City, Vietnam; ^2^ Institute of Biotechnology and Food Technology, Industrial University of Ho Chi Minh City, Ho Chi Minh City, Vietnam; ^3^ Faculty of Food Technology, Saigon Technology University, Ho Chi Minh City, Vietnam; ^4^ Institute of Advanced Technology, Vietnam Academy of Science and Technology, Ho Chi Minh City, Vietnam

**Keywords:** anthocyanin, deep eutectic solvent, nanoliposome, green extraction, black rice, stability kinetics

## Abstract

This study investigated the recovery and nanoliposomal formulation of anthocyanin-rich extracts from black rice using choline chloride-based deep eutectic solvents (DESs). Choline chloride/1,2-propanediol (DES2) produced the highest anthocyanin content, 85.5 ± 3.4 mg l^−1^, after optimization, compared with 25.7 ± 2.1 mg l^−1^ obtained by conventional ethanol extraction. High-resolution QTOF-MS/MS enabled the tentative assignment of cyanidin 3-*O*-hexoside-type and peonidin 3-*O*-hexoside-type compounds based on accurate precursor masses and characteristic product ions; authentic-standard confirmation was not performed. The DES2 extract showed greater apparent thermal stability than the control extract, with estimated half-lives of 30.0 and 16.0 days, respectively, at 45°C. Anti-oxidant activity was observed in DPPH and ABTS assays, with IC50 values of 0.19 ± 0.06 and 0.16 ± 0.08 mg ml^−1^, respectively. Preliminary antibacterial activity against *Escherichia coli* was detected under the tested agar-diffusion conditions, whereas activity against *Staphylococcus aureus* was limited. Nanoliposomal encapsulation achieved an encapsulation efficiency of 73.8 ± 4.3%, a Z-average diameter of 201.3 nm and 89.83 ± 0.65% anthocyanin retention after 28 days at 4°C. The formulation produced a larger inhibition zone against *E. coli* than the free extract under the assay conditions. These findings support further investigation, although release kinetics, pharmaceutical efficacy and complete-process sustainability remain unestablished.

## 1. Introduction

Black rice (*Oryza sativa* L.) is a pigmented rice variety with a long history of cultivation, distinct from common white rice. Originating in Asia, genetic evidence suggests it shares a common ancestry with other coloured varieties, having been cultivated for approximately 9000 years. While primarily grown and consumed in Asian regions, black rice is increasingly recognized globally for the nutritional value of its bran layer [1]. Beyond basic nutrients like starch and protein, the outer grain layers are rich in anthocyanins (ACNs), natural pigments responsible for its deep purple-black colour and associated with various reported biological activities [2]. Owing to these properties, it has historically been revered as ‘longevity rice’ and is now considered a valuable functional food ingredient [3]. ACNs are a large and diverse group of water-soluble pigments within the flavonoid family, with over 600 distinct compounds identified in nature [4]. These compounds not only are significant as natural colourants, capable of producing hues from red to purple depending on pH, but also possess a broad spectrum of various reported biological activities. Research has extensively documented their roles in anti-oxidant defence, allergy prevention, radiation protection, anti-inflammatory responses, antimicrobial action and cardiovascular protection [5].

Despite their potential, the efficient recovery of ACNs from plant matrices presents challenges. Conventional extraction methods predominantly rely on organic solvents such as methanol or ethanol–water mixtures. While techniques like ultrasound-assisted extraction have been employed to improve yield through process optimization [6], and microwave-assisted extraction has shown efficacy by rapidly heating and disrupting cellular structures [7,8], these methods often involve volatile organic solvents or high energy inputs that may compromise environmental sustainability.

To address these limitations, deep eutectic solvents (DESs) have emerged as alternative extraction media for the extraction of bioactive compounds [9,10]. DESs have been reported to exhibit tunable physico-chemical properties and, depending on their composition, may offer favourable environmental or toxicological profiles. These properties require evaluation for each specific formulation [11–14]. Recent studies indicate that DESs can significantly enhance extraction yields compared to conventional solvent systems [15,16]. For instance, choline chloride-based DESs demonstrated superior efficiency in extracting ACNs from grape skins compared to acidified methanol [17], and similar systems improved the recovery of mulberry ACNs by over 20% relative to traditional organic solvents [18]. However, the industrial application of extracted ACNs is often hindered by their natural instability. These molecules degrade rapidly when exposed to adverse conditions such as varying pH, high temperatures, oxygen, light and metal ions [19]. To mitigate this, microencapsulation technologies are widely adopted to protect the bioactive core [20]. Various encapsulation strategies have been explored, including microcapsules [21,22], nanoemulsions [23–25], microemulsions [26,27], Pickering emulsions [28] and hydrogels [29]. Nanoliposomes have been investigated as carriers for improving the physical stability and accessibility of water-soluble bioactive compounds [30,31]. Composed of a phospholipid bilayer [32], nanoliposomes exhibit exceptional biocompatibility, mimicking cell membranes to facilitate fusion and penetration into microorganisms [33]. This unique structure not only enhances the cellular uptake and tissue distribution of bioactive compounds but also offers advantages such as low toxicity, high flexibility, biodegradability and self-assembly capabilities [24].

By encapsulating water-soluble ACNs within their aqueous core, nanoliposomes effectively shield the pigments from environmental degradation factors like light and pH, thereby significantly prolonging their bioavailability [32,34,35]. Although DESs are excellent extraction media and nanoliposomes are effective delivery systems, the integration of DES-based extraction and nanoliposomal formulation for black rice ACNs remains limited. Most existing literature focuses on either extraction optimization or stabilization separately. Consequently, an integrated strategy that combines DES-assisted extraction with subsequent nanoliposomal stabilization for black rice ACNs remains largely unexplored.

We hypothesized that a choline chloride-based DES, when combined with ultrasound assistance, would produce higher measured ACN contents than the conventional ethanol-based extraction under the investigated conditions owing to enhanced hydrogen-bonding solubilization, and that subsequent nanoliposomal encapsulation of the resulting extract would improve its colloidal and storage stability relative to the free extract. To our knowledge, this study is among the few studies to integrate DES-based extraction with nanoliposomal encapsulation of ACN-rich extracts from black rice within a single workflow.

In this study, we investigated the extraction of ACNs from black rice using choline chloride-based DESs. The physico-chemical properties of the optimal solvent and extract were characterized. Subsequently, the DES-enriched extract was encapsulated into nanoliposomes to evaluate its physico-chemical characteristics and ACN retention during storage. Finally, the anti-oxidant and antibacterial properties of the DES2 extract and a conventional extract were evaluated under the assay conditions used.

## 2. Material and methods

### 2.1. Materials and chemicals

Black rice (*O. sativa* L.) used in this study was the Phuc Tho Black cultivar, harvested in March 2024 from the Red River Delta, Hanoi, Vietnam. The botanical identity and cultivar authentication were verified by the VNM Herbarium, Institute of Tropical Biology, Vietnam Academy of Science and Technology, Ho Chi Minh City, Vietnam. The intact grains were sun-dried, dehusked and stored in airtight polyethylene bags at 4°C prior to processing. Before extraction, the black rice grains were pulverized using a high-speed laboratory grinder and sieved through a 60-mesh sieve to obtain a uniform particle size (<250 µm).

Glycerol, 1,2-propanediol, acetic acid, l-lactic acid, ethanolamine, citric acid, choline chloride (ChCl) and ethanol were obtained from Xilong (China). Trolox (6-hydroxy-2,5,7,8-tetramethylchroman-2-carboxylic acid) and DPPH (2,2-diphenyl-1-picrylhydrazyl) were obtained from Sigma-Aldrich Pty Ltd (USA). All chemicals were of analytical grade and used directly without further purification.

### 2.2. Preparation and characterization of deep eutectic solvents

A series of DESs were fabricated by employing ChCl as the hydrogen bond acceptor (HBA). Various hydrogen bond donors (HBDs) were selected, including polyols (glycerol, propane-1,2-diol), organic acids (citric, acetic, ascorbic and lactic acids) and ethanolamine. The synthesis followed a thermal mixing procedure where the HBA and HBD were combined at a fixed molar ratio of 1:1. The mixtures were subjected to continuous agitation (300 rpm) in a water bath maintained at 80°C for approximately 2 h until a stable, clear and homogeneous liquid phase was achieved. The pH of the resulting solvents was measured using a pH meter (SevenExcellence S500, Mettler-Toledo, LLC, OH, USA) calibrated at 25°C. To characterize the functional groups and examine possible intermolecular interactions in the prepared systems, Fourier transform infrared (FTIR) spectroscopy was performed using a Jasco FT/IR-6600 spectrometer (USA). The spectra were recorded in the wavenumber range of 4000–400 cm^−1^ to analyse shifts in characteristic functional groups of the HBAs, HBDs and the resulting liquid systems.

### 2.3. Extraction process

The extraction procedure was conducted as follows: a 1.0 g sample of black rice was placed into a 50 ml conical flask containing 15 ml of a mixture of DES and distilled water (30%, v/v). The mixture was shaken for 30 s to ensure thorough mixing and was subsequently subjected to ultrasound treatment at 60°C and 3.0 W g^−1^ for 30 min using an ultrasonic bath (500W, TUC, LABO-HUB, Shanghai, China). Next, the mixture was centrifuged at 5000 r.p.m. for 5 min to collect the supernatant, which was then filtered through a 0.45 µm membrane. The concentration of ACN was determined using differential pH methods.

Four variables were controlled during the extraction using DES to evaluate their impact on ACN content: liquid-to-solid ratio (10–30 ml g^−1^), water content (10–40%), extraction time (10–60 min) and ultrasound power (3–6.0 W g^−1^). When one factor was changed, the values of the other variables were kept constant to accurately assess the ACN content. A control experiment was conducted similarly using a mixture of water/ethanol/acetic acid solvent (50:49:1) in previous work [36]. All experiments were performed in triplicate to calculate the average and standard deviation.

### 2.4. Quantification and identification of anthocyanins

#### 2.4.1. Total anthocyanin content quantification

The total ACN content was determined using the differential pH method with slight adjustments [37,38]. Briefly, 2.0 ml of the extracted sample was thoroughly mixed with 8 ml of buffer solution. This mixture was then diluted approximately 10-fold using a pH 1 buffer solution (0.025 M KCl, 0.1 M HCl). The same procedure was repeated with a pH 4.5 buffer solution (acetate buffer). Absorbance values were measured sequentially at 520 and 700 nm for both pH conditions using a Genesys 10s UV–Vis spectrophotometer (USA). The absorbance difference (*A*) was calculated using equation (2.1):

(2.1) $$A=(A_{520}-A_{700})pH_{1}-(A_{520}-A_{700})pH_{4.5}.$$

The total ACN content (mg l^−1^) was subsequently determined using equation (2.2):

(2.2) $$Content\;of\;ACNs=\frac{A\times MW\times DF\times 1000}{\left(E\times W\right)}\;\left(mg\;l^{-1}\right).$$

In these equations, *A* is the difference in absorbance; MW is the molecular weight of cyanidin 3-glucoside (C3G), 449.2 g mol^−1^; DF is the dilution factor; *E* is the molar extinction coefficient (26.900 l mol^−1^ cm^−1^); *W* is the path length of the cuvette (cm).

#### 2.4.2. Identification of individual anthocyanin compounds in extract

To tentatively assign the major anthocyanins in the DES2 extract, high-resolution liquid chromatography quadrupole time-of-flight tandem mass spectrometry (LC-QTOF-MS/MS) analysis was performed using an Agilent 1290 Infinity UHPLC system coupled to an Agilent 6545 LC/Q-TOF mass spectrometer. Chromatographic separation was performed on an Agilent ZORBAX Eclipse Plus C18 column (2.1 × 50 mm, 1.8 µm) using a mobile phase consisting of (i) water with 0.1% formic acid and (ii) acetonitrile/methanol under gradient elution at 0.4 ml min^−1^. The mass spectrometer was operated in positive electrospray ionization (ESI+) mode with a fragmentor voltage of 150 V. Full-scan MS1 spectra were acquired over *m*/*z* 50–650, and collision-induced dissociation was performed at stepped collision energies of 10, 20 and 40 eV to generate diagnostic product-ion spectra to support tentative structural assignment. Precursor and product ions were assigned by accurate mass matching (mass error < 2 ppm) against theoretical exact masses calculated from the corresponding molecular formulae, and extracted-ion chromatograms were generated for each precursor mass to verify peak purity. Compound assignments were based on accurate precursor masses, characteristic product ions and comparison with reported ACN fragmentation patterns. Because authentic-standard co-injection was not performed, the assignments are considered tentative rather than definitive identifications.

### 2.5. Evaluation of anthocyanin stability in the extract

#### 2.5.1. Effect of pH

To evaluate the stability of ACN in the extract under different pH conditions, a series of test tubes containing 5 ml of the extract were adjusted to pH values ranging from 1.0 to 9.0. The test tubes were then covered with aluminium foil and maintained at a temperature of 25°C for 30 min, and the remaining ACN content (%) was measured using the differential pH method.

#### 2.5.2. Kinetic modelling

Kinetic modelling of ACN degradation was performed following established methodologies, notably drawing from the approach described by Marangoni Júnior *et al.* [39]. The stability of the DES2 extract was assessed at 4, 25 and 45°C at pH 1 in the absence of light. Residual ACN content was measured at 0, 7, 14, 21 and 28 days. The thermal degradation of ACNs under isothermal conditions is generally understood to follow first-order kinetics. The integrated form of this reaction rate is mathematically expressed in equation (2.3):

(2.3) $$\begin{array}{r} \ln\frac{C_{t\;}}{C_{0}}=-kt, \end{array}$$

where *C_t_* represents the total ACN concentration measured at time *t* and *C*_0_ indicates the initial anthocyanin concentration at *t* = 0.

The influence of temperature on the thermal degradation rate constant (*k*) for these reactions can be described using the Arrhenius equations, presented in equation (2.4):

(2.4) $$\begin{array}{r} k=k_{0}{\text{e}}^{-E/RT}, \end{array}$$

where *k*_0_ is the pre-exponential factor, *E* represents the activation energy for the degradation process (J mol^−1^), *R* is the gas constant (J mol^−1^ K^−1^), and *T* is the temperature during the process (K).

The half-life *t*_1/2_ of the degradation process is expressed in equation (2.5):

(2.5) $$\begin{array}{r} t_{1/2}=\frac{\ln2}{k}, \end{array}$$

where *k* is the kinetic rate constant (day^−1^).

#### 2.5.3. Thermodynamic analysis

The activation enthalpy (∆*H*), the apparent activation free energy (∆*G*) and the entropy (∆*S*) were determined using equations (2.6)–(2.8), according to previous works [39,40]:

(2.6)ΔH=E−R×T,(2.7)ΔG=−R×Tln⁡k×hkB×T,(2.8)ΔG=ΔH−T×ΔS,

where *E* denotes the activation energy for ACN thermal degradation (J mol^−1^), *T* is the absolute temperature (K), *k* represents the kinetic rate constant (s^−1^), *R* is the ideal gas constant (8.314 J mol^−1^ K^−1^), *k_B_* is the Boltzmann constant (1.3806 × 10^−23^ J K^−1^), and *h* is Planck's constant (6.6262 × 10^−34^ J s).

The rate constants obtained from the first-order model were initially expressed in day^−1^. For thermodynamic calculations using equation (2.7), the values were converted to s^−1^ by dividing by 86 400.

### 2.6. Preparation of nanoliposomes loaded with deep eutectic solvent extract

#### 2.6.1. Preparation of NLDES2

Nanoliposomes loaded with the ACN-rich DES2 extract (hereafter referred to as NLDES2) were prepared using a modified solvent-injection method, adapted from previous reports [41,42], to ensure efficient encapsulation and consistent particle characteristics. The process commenced with the preparation of the lipid phase, involving the accurate dissolution of 0.25 g of lecithin in 10 ml of dichloromethane (DCM). Concurrently, the aqueous phase was prepared using the optimized DES2 extract, which contained ACNs at a concentration of 0.08 mg ml^−1^. The lipid phase was then slowly injected, dropwise, into the 100 ml aqueous phase, and the entire system was subjected to continuous magnetic stirring at 500 r.p.m. for 30 min.

Following this injection, the resulting nascent liposome suspension was heated to 50°C for 30 min on the stirring platform. This critical step ensured the complete and controlled evaporation of DCM.

Subsequently, 1.2 ml of Tween 80 was introduced, and the entire system was subjected to continuous magnetic stirring at 500 r.p.m. This stirring facilitated the dispersion and stabilization of the formed nanoliposomes. Finally, to achieve further refinement in particle size and enhance the homogeneity of the dispersion, the suspension underwent ultrasonication. This final homogenization step was performed for 30 min at a power of 300 W, using a 500 W ultrasonic bath (TUC, LABO-HUB, Shanghai, China), ultimately yielding the NLDES2 suspension for subsequent characterization and storage testing.

The encapsulation efficiency (EE) of the nanoliposomes was determined using a previous report [43], with slight adjustments. The procedure was as follows: 2.0 ml of the nanoliposome sample containing ACN was placed in a dialysis bag (MWCO of 3.5 kDa) and immersed in 50 ml of distilled water, stirring at 100 r.p.m. for 2 h. The ACN content that diffused through the membrane was determined using the differential pH method. The experiment was repeated three times for each extract. The EE was calculated according to equation (2.9):

(2.9) $$\begin{array}{r} EE\;\left(\%\right)=\frac{\left(C_{t}-C_{i}\right)}{C_{t}}\times 100, \end{array}$$

where *C_t_* represents the initial amount of ACN used for the preparation of the nanoliposomal formulation and *C_i_* represents the amount of ACN measured outside the dialysis bag after dialysis.

#### 2.6.2. Storage stability of NLDES2

The physical and encapsulation stabilities of the optimized nanoliposomes (NLDES2) were evaluated over a 28-day storage period. Freshly prepared NLDES2 suspensions were placed in sealed amber glass vials to prevent light-induced degradation and stored at two different conditions: 4°C (refrigeration) and 25°C (room temperature). At specific time intervals (days 0, 7, 14, 21 and 28), aliquots were withdrawn for analysis.

To assess physical stability, the mean particle size (Z-average), polydispersity index (PDI) and zeta potential of the nanoliposomes were measured using a dynamic light scattering (DLS) instrument. Prior to DLS measurements, the samples were appropriately diluted with deionized water to avoid multiple scattering effects.

To evaluate encapsulation stability, the ACN retention rate was monitored. The concentration of retained ACNs within the liposomes at each specific time point was quantified following the same analytical procedure used for determining the EE (using the same differential pH procedure described in §2.4.1). The ACN retention was calculated as the percentage of the initial encapsulated ACN content remaining at storage time (*t*) using the following equation:

(2.10) $$\begin{array}{r} Retention\;rate\;\left(\%\right)=\left(C_{t}/C_{0}\right)\times 100, \end{array}$$

where *C_t_* is the concentration of encapsulated ACNs at storage time *t* (days 7, 14, 21 and 28) and *C*_0_ is the initial concentration of encapsulated anthocyanins at day 0. All experiments and measurements were performed in triplicate, and the results were expressed as mean ± standard deviation (*n* = 3).

### 2.7. Determination of some physico-chemical properties

The morphology and surface structure of the raw materials before and after extraction indicate the structural resilience of the cellulose layer against solvent attack. This information can provide insights into the interaction between DES and the surfaces of the raw materials. To analyse the morphology of the raw material samples before and after extraction, scanning electron microscopy (SEM) using a Hitachi S4800 (Japan) was employed. Before analysis, the samples were treated with a glutaraldehyde solution for 2 h, followed by preservation at −4°C for 5 min. The dehydration process was performed using ethanol and vacuum drying, followed by sputtering a thin layer of carbon and gold to enhance conductivity. The samples were imaged at different magnifications of 20, 5 and 1 µm.

Some physico-chemical properties of the solvent and extract were analysed using FTIR spectroscopy, DLS and transmission electron microscopy (TEM). The infrared spectrum was obtained using a Jasco FT/IR-6600 spectrometer (USA) to identify the characteristic vibrations of functional groups in the DES, the extract and the nanosystem. Parameters, such as the hydrodynamic particle size (Z-average), zeta potential and vesicle morphology, were measured to clarify the characteristics of the nanosystem. Specifically, a HORIBA SZ-100 DLS device (Japan) was used to determine the average hydrodynamic particle diameter (Z-average) and zeta potential in the nanoliposome system, while TEM (HT 7700, Hitachi Ltd, Japan) was conducted to observe the vesicle shape of the system.

### 2.8. Bioactivity assays

#### 2.8.1. DPPH radical-scavenging assay

The anti-oxidant capacity of the samples was determined spectrophotometrically through their ability to scavenge DPPH radicals, following established protocols [44,45]. A Trolox standard curve was constructed using Trolox solutions at appropriate concentrations (e.g. 10–40 µg ml^−1^). Briefly, 0.1 ml of each standard solution was mixed with 2.4 ml of a 0.2 mM DPPH solution to achieve a final reaction volume of 2.5 ml. The mixtures were incubated in a rotary shaker at 30 ± 2°C and 250 r.p.m. for 30 min in the dark.

For sample analysis, stock solutions (12.5 mg ml^−1^) of the DES2-free, DES2 extract and NLDES2 underwent twofold serial dilutions to obtain a concentration series ranging from 12.5 to 0.78125 mg ml^−1^. Subsequently, 0.1 ml of each dilution was mixed with 2.4 ml of the DPPH solution, resulting in final test concentrations of 0.5–0.03125 mg ml^−1^. The mixtures were incubated under identical conditions to the standards.

The maximum absorbance was measured at 517 nm using a UV–visible spectrophotometer, with methanol/ethanol serving as the blank. All assays were performed in triplicate. Anti-oxidant activity was quantified as the percentage of radical scavenging (*I*), calculated using equation (2.11) and expressed as the IC50 value:

(2.11) $$\begin{array}{r} I(\%)=\frac{A_{0}-A_{sample}}{A_{0}}\times 100, \end{array}$$

where *A*_0_ and *A*_sample_ are the absorbance of the control (DPPH solution without sample) and the tested sample, respectively.

#### 2.8.2. ABTS radical-scavenging assay

The ABTS (2,2′-azinobis(3-ethylbenzothiazoline-6-sulfonic acid)) radical-scavenging activity was evaluated following established protocols [46]. Initially, the ABTS radical cation (ABTS•^+^) was generated by reacting a 7 mM ABTS aqueous solution with 2.45 mM potassium persulfate (1:1, v/v) in the dark for 12–16 h. Prior to the assay, the solution was diluted with ethanol to reach an absorbance of 0.70 ± 0.02 at 734 nm.

The preparation procedures for the Trolox standard curve and the twofold serial dilutions of the samples (0.1 ml sample mixed with 2.4 ml reagent) were identical to those described for the DPPH assay (§2.8.1), substituting the DPPH reagent with the diluted ABTS•^+^ solution.

To ensure consistent comparison among samples, all mixtures were incubated for 30 min in the dark at room temperature before absorbance measurement. This incubation period was applied uniformly to the DES2 extract and NLDES2 samples and was not intended to measure or demonstrate release kinetics, differing from the standard 6 min protocol reported for the assay. The absorbance was measured at 734 nm. The scavenging percentage and IC50 values were calculated analogously to the DPPH assay using equation (2.11).

#### 2.8.3. Antibacterial activity

In this study, we selected two bacterial strains, *Staphylococcus aureus* and *Escherichia coli*, to evaluate the inhibitory effects of the DES2 extract, a positive control (Control (+)) and a negative control (Control (−)). Chloramphenicol (1000 µg ml^−1^) was used as Control (+); the corresponding solvent/vehicle without ACN extract was used as Control (−). The procedure was conducted following the agar well diffusion method [47]. The bacterial cultures were standardized to a concentration of approximately 10^6^ CFU ml^−1^. The culture medium was nutrient agar. After preparing the agar medium and allowing it to solidify, the bacterial suspension was evenly spread over the surface of the agar. Each extract or formulation was tested at a nominal concentration of 100 mg ml^−1^, and 10 µl of sample was added to each well. Chloramphenicol was added at 10 µl and 1000 µg ml^−1^ as the positive control. The agar plates were incubated at 37°C for approximately 24 h. After the incubation period, the diameter of the inhibition zone was measured using calipers to assess antibacterial activity.

### 2.9. Data analysis

All experimental data were expressed as the mean ± standard deviation (s.d.) from three independent replicate measurements. Statistical comparisons were performed using unpaired Student's *t*-tests and one-way analysis of variance (ANOVA) to assess significant differences between groups. A *p*-value of less than 0.05 (*p* < 0.05) was considered statistically significant. Data calculation and initial statistical processing were conducted using Microsoft Excel, while graphical representation and advanced statistical analyses were performed using OriginLab Origin 2024 software (OriginLab Corporation, USA).

## 3. Results and discussion

### 3.1. Physico-chemical properties of deep eutectic solvents

All prepared DESs were water soluble and had densities higher than that of water in the range of 1.09–1.45 g ml^−1^. Regarding pH values, two polyalcohol-based DESs (DES1 and DES2) exhibited a neutral pH environment (table 1). In contrast, DESs containing organic acids (DES3–DES6) showed relatively low pH values. This observation aligns well with previous reports [48,49]. DES7 bearing NH_2_ group exhibited a strongly basic pH.

**Table 1.** Some properties of the DESs.

DES	HBD	mole ratio	density (g ml^−^^1^)	pH
DES1	glycerol	1:1	1.12 ± 0.01	7.09 ± 0.11
DES2	1,2-propanediol	1:1	1.09 ± 0.04	7.03 ± 0.20
DES3	citric acid	1:1	1.35 ± 0.02	1.68 ± 0.10
DES4	acetic acid	1:1	1.31 ± 0.03	1.71 ± 0.12
DES5	ascorbic acid	1:1	1.42 ± 0.09	1.67 ± 0.20
DES6	lactic acid	1:1	1.37 ± 0.02	1.57 ± 0.11
DES7	ethanolamine	1:1	1.22 ± 0.02	10.24 ± 0.32

Values are expressed as mean ± standard deviation (*n* = 3).

The physico-chemical properties of the synthesized DESs were comprehensively characterized using UV–visible and FTIR spectroscopies (figure 1). Specifically, UV–visible spectroscopy was employed to investigate the absorption characteristics. A consistent spectral profile was observed across all DES formulations, which were characterized by prominent and distinct absorption peaks centred around 280 nm. These characteristic absorption bands are primarily attributed to electron transitions occurring within the molecular components of the DESs, notably involving *n* → π* and *n* → σ* transitions [9].

**Figure 1.**
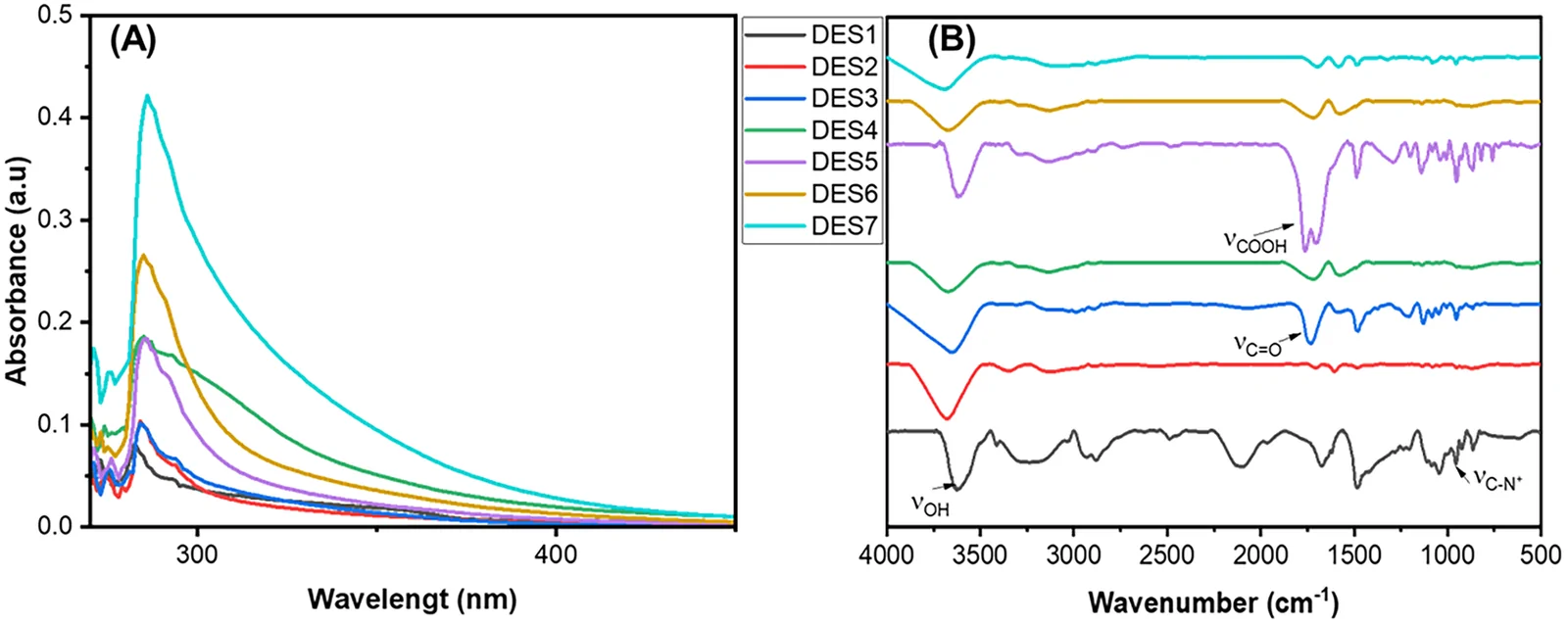
(A) UV absorption spectra and (B) FTIR spectra of DESs.

The FTIR spectra, presented in figure 1B and further elaborated in figures S1–S7 in the supplementary material, were used to characterize the functional groups and spectral changes associated with interactions between ChCl and the HBDs. Significant variations were observed within the fingerprint region (2000–500 cm^−1^) across different DES formulations, which can be attributed to their distinct structural compositions. Common absorption bands observed for all DESs included a broad band in the 3600–3200 cm^−1^ region, characteristic of –OH stretching vibrations. Bands corresponding to C=O stretching vibrations appeared between 1730 and 1650 cm^−1^, while C–H stretching vibrations were evident in the 3000–2500 cm^−1^ range. Additionally, C–O stretching vibrations were assigned to the region between 1112 and 1000 cm^−1^. Furthermore, all DESs consistently displayed absorption bands at approximately 1480 cm^−1^, indicative of the bending vibrations of the –OH group, and around 950 cm^−1^, attributed to the C–N^+^ group. A distinct sharp peak at 1080 cm^−1^ was specifically observed for DES1 and DES2, both derived from polyol-based HBDs, corresponding to the C–OH stretching vibration. Overall, the observed spectral features were consistent with intermolecular interactions between ChCl and the selected HBDs and supported the formation of the prepared liquid ChCl-based systems under the conditions used. However, FTIR and UV–visible data alone do not definitively establish the eutectic state.

### 3.2. Solvent screening

The extraction capability of ACNs using different DESs depends on the properties of the DES, including polarity, electrostatic interactions, hydrogen-bonding potential and donor–acceptor interactions. The DESs were screened to identify a candidate solvent for subsequent extraction experiments. Significant differences in the measured ACN contents were observed among the solvent systems (figure 2). The control sample yielded an ACN concentration of 18.4 ± 1.5 mg l^−1^. Among the fabricated DESs, DES2 produced the highest measured ACN content, with an ACN content of 66.6 ± 2.2 mg l^−1^, followed closely by DES4. Specifically, the measured ACN content of DES2 was approximately 3.6 times that of the control sample, while DES4 showed a measured ACN content approximately twice that of the control. The other DESs produced lower measured ACN contents compared to the control. These findings suggest that the measured ACN content depends on the composition and structure of the DESs. Under the screening conditions used in this study, the acid-based DESs yielded lower measured ACN contents than DES2 when extracting ACNs from black rice, despite their reported effectiveness in extracting ACNs from blueberries [50,51]. As a result, DES2 was selected for subsequent experiments because it produced the highest measured ACN content under the screening conditions.

**Figure 2.**
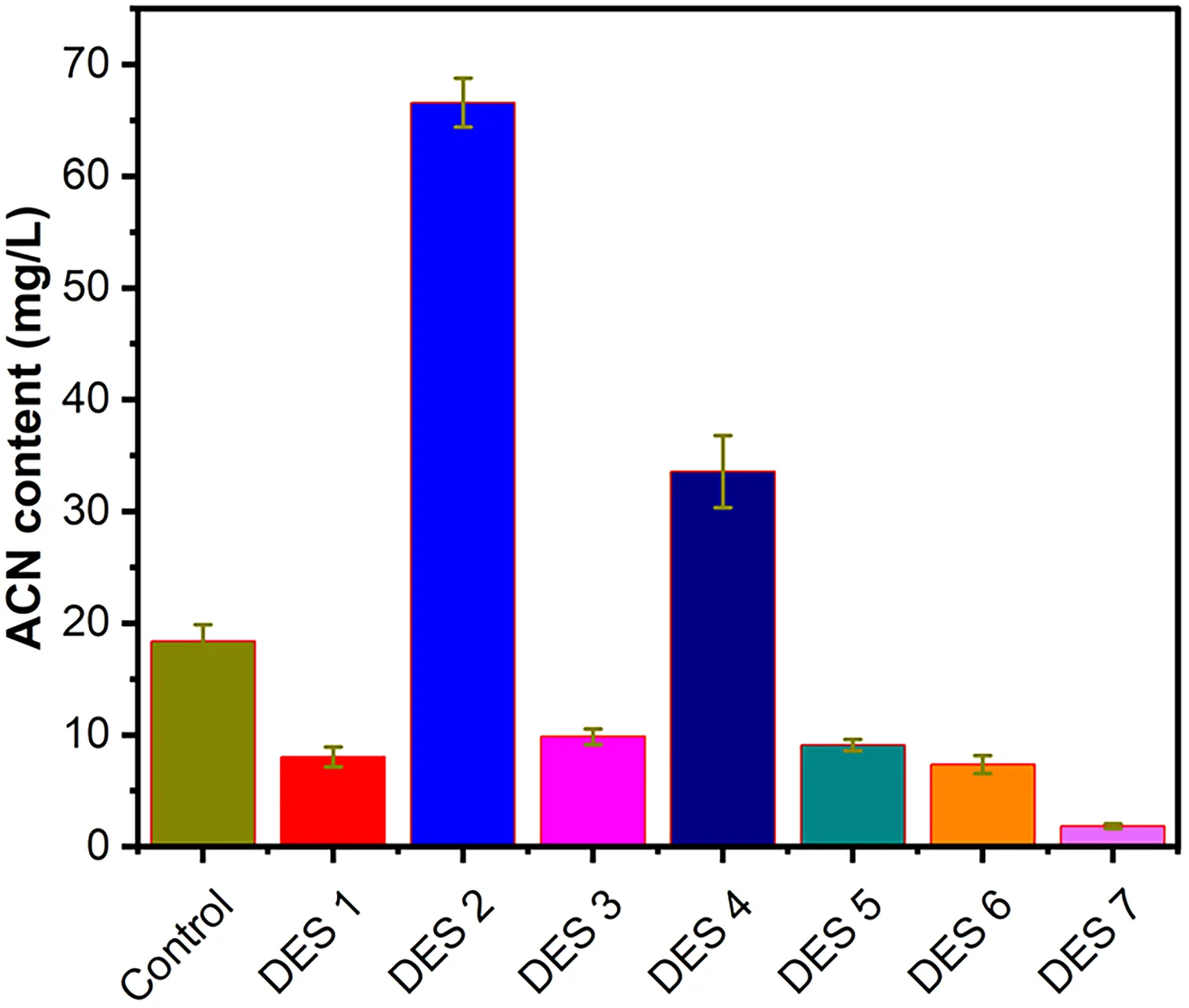
Solvent screening to identify the optimal solvent under the following conditions: solvent-to-material ratio of 20:1 (v/w), water-to-DES ratio of 30% (v/v), ultrasound power of 3 W g^−1^ and ultrasound duration of 10 min.

### 3.3. Optimization of anthocyanin extraction using DES2

#### 3.3.1. Liquid-to-solid ratio

The solvent-to-raw-material ratio was evaluated to determine a suitable ratio for the extraction process. As shown in figure 3A, the ratio significantly affected the measured ACN content. For DES2, the ACN content increased from (27.6 ± 1.4) mg l^−1^ to reach a maximum of (83.3 ± 2.4) mg l^−1^ at a ratio of 10:1, after which it gradually decreased. In contrast, the control treatment showed relatively little variation, with the highest measured ACN content observed at a ratio of 25:1. Under the conditions investigated, the 10:1 liquid-to-solid ratio was therefore selected for subsequent experiments with DES2. At low solvent-to-solid ratios, limited solvent capacity and possible solute saturation may reduce the measured ACN content obtained. Conversely, excessive solvent use increases operating costs and may reduce solute recovery during subsequent processing.

**Figure 3.**
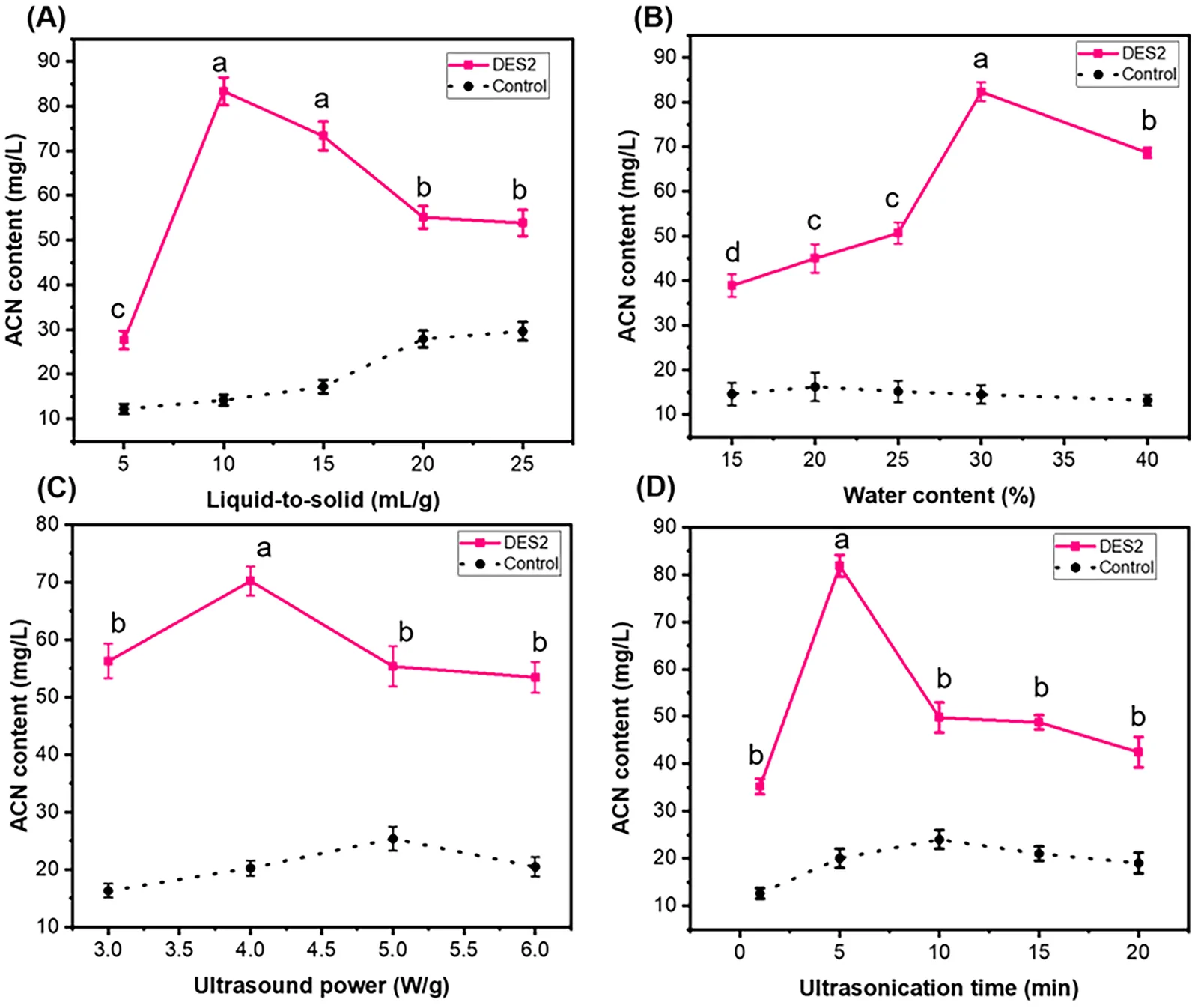
Factors affecting the measured ACN content obtained using DES2: (A) liquid-to-solid ratio, (B) water content, (C) ultrasound power, and (D) ultrasonication time. Each value is expressed as mean ± s.d. (*n* = 3), which means that different superscripts at the data points indicate a significant difference (*p* < 0.05).

#### 3.3.2. Water content

The water content in DES is a crucial factor influencing the measured ACN content of bioactive compounds from biomass, primarily through the polarity of the solvent [52]. In this study, the water ratio in DES2 was adjusted from 10 to 40% to evaluate its impact on measured ACN content (figure 3B). The results indicated that the ACN content increased as the water ratio rose from 10% to 30%; however, the measured ACN content decreased at 40% water content relative to the maximum observed at 30%. This decrease may be associated with changes in solvent polarity, viscosity, hydrogen-bonding interactions or ACN stability; these possibilities were not directly tested. As the water content increases, the polarity of the DES also rises, improving the solubility and interaction of biological compounds with the solvent. Additionally, the water content affects the surface tension and viscosity of the DES, which enhances mass transfer and the measured ACN content obtained [53]. Therefore, a water content of 30% was determined to be optimal for the ACN extraction process using DES2. Previous studies reported similar effects of water content on the extraction efficiency of polyphenols from biomass [54] or on the yields of adenosine and cordycepin from *Cordyceps* mushroom powder [55].

#### 3.3.3. Ultrasound power

Ultrasound power is a critical factor influencing ACN content during extraction processes. Ultrasound generates high-frequency sound waves, causing cavitation and the formation of tiny bubbles in the solution [56]. The work highlights the critical role of selecting the optimal ultrasound power to maximize ACN content during extraction, thereby improving both the efficiency and quality of ACN-containing products. As shown in figure 3C, ACN content increased significantly from 56.3 ± 2.5 to 70.2 ± 3.2 mg l^−1^ as ultrasound power was raised from 3 to 4 W g^−1^. However, further increases in power to 5 and 6 W g^−1^ resulted in a decline in ACN extraction. This is likely owing to the intense energy released from collapsing bubbles at higher ultrasound powers, which elevates local pressure and temperature, disrupting cellular structures and enhancing ACN release. While moderate ultrasound power amplifies this effect, excessively high power may cause ACN degradation owing to elevated local temperatures, thereby reducing its concentration in the final product. Therefore, 4 W g^−1^ was selected for subsequent experiments.

#### 3.3.4. Ultrasonication time

Figure 3D illustrates the dependence of ACN content on sonication time. The highest anthocyanin content was obtained at 5 min (81.8 ± 3.2 mg l^−1^), while the lowest was recorded at 10 min (49.7 ± 2.3 mg l^−1^). According to the ultrasound mechanism, longer sonication times lead to greater heating in the solvent, which causes increased cavitation and bubble collapse, potentially promoting ACN degradation. This leads to the generation of free radicals and a decrease in the measured ACN content. Based on the data obtained, we selected a sonication time of 5 min for the subsequent experiments.

Based on the conducted optimization, we determined the best conditions for the extraction as follows: a solvent-to-raw-material ratio of 10:1, a water content of 30%, an ultrasound power of 4.0 W g^−1^ and a sonication time of 5 min. The experiment was repeated three times under these conditions, resulting in an ACN content of 85.5 ± 3.4 mg l^−1^. In contrast, the extract using a mixture of ethanol and water yielded an ACN content of 25.7 ± 2.1 mg l^−1^. This result demonstrates the higher measured ACN content obtained under the optimized DES2 conditions.

To provide a preliminary quantitative comparison of material use, a process mass intensity (PMI) analysis was performed for the extraction step using identical solvent-to-solid ratios and ultrasound conditions (§3.3.4). The DES2-based protocol required a PMI of approximately 13 600 mg input per mg of measured ACN-equivalent content, compared with approximately 38 700 mg mg^−1^ for the conventional ethanol-based extraction. Under the conditions investigated and within the defined system boundaries, this lower PMI was associated with the higher measured ACN content obtained using DES2. However, the PMI comparison was limited to the extraction step and does not by itself establish the environmental sustainability of the complete extraction-to-formulation process. Energy consumption and other relevant factors, including solvent recovery, water use, solvent toxicity, waste treatment, the use of dichloromethane during liposome preparation and scale-up performance, were not comprehensively assessed. DES formation may proceed without a separate purification step and has been described as having no associated process waste under the specific preparation conditions reported previously [57]. Nevertheless, solvent recyclability was not experimentally evaluated in the present study. Although ChCl-based DES systems have been reported to be reused for three to five extraction cycles without a significant loss of efficiency [58], these findings support only the feasibility of further investigation and should not be interpreted as evidence of recyclability or closed-loop operation in the present system.

While the one-factor-at-a-time (OFAT) approach employed in this study successfully established the baseline operational parameters and demonstrated the superior measured ACN content obtained using DES2, it is important to acknowledge that this method precludes the assessment of complex interactive effects between extraction variables. As a preliminary investigation, the current OFAT optimization provides a solid foundation. Future studies should implement statistically rigorous multivariate optimization designs, such as response surface methodology, to further scale up, model variable interactions and comprehensively optimize this DES-based extraction protocol for future process development.

### 3.4. Characterization of black rice extract and identification of anthocyanins

The microstructural transformations of black rice powder before and after extraction were systematically investigated using SEM (figure 4). As shown in figure 4A, the untreated black rice flour exhibited a dense, heterogeneous architecture characterized by agglomerated organic components. At higher resolution (1 µm), well-defined, angular and polyhedral starch granules remained completely intact and firmly embedded within the cellular matrix, serving as a primary physical barrier to active compound release. Following conventional ethanol extraction (figure 4B), the flour retained its general particulate framework; however, superficial erosion and localized micro-cracks emerged on the surface owing to the partial leaching of soluble constituents. In sharp contrast, the residue subjected to DES2-assisted extraction (figure 4C) displayed marked structural disruption. The residue showed greater surface erosion and pore formation, revealing a more porous matrix populated by numerous etched micropores. The observed morphological changes may be consistent with enhanced matrix disruption and removal of soluble components during DES2-assisted ultrasound extraction. The observed surface disruption may have contributed to reduced mass-transfer resistance during extraction; however, changes in cell-wall permeability were not directly measured. The marked decolorization of the DES2 residue compared with the alcohol-extracted counterpart, which retained a distinct purple hue (supplementary material, figure S8), is consistent with greater removal of ACN-related pigments during DES2-assisted extraction.

**Figure 4.**
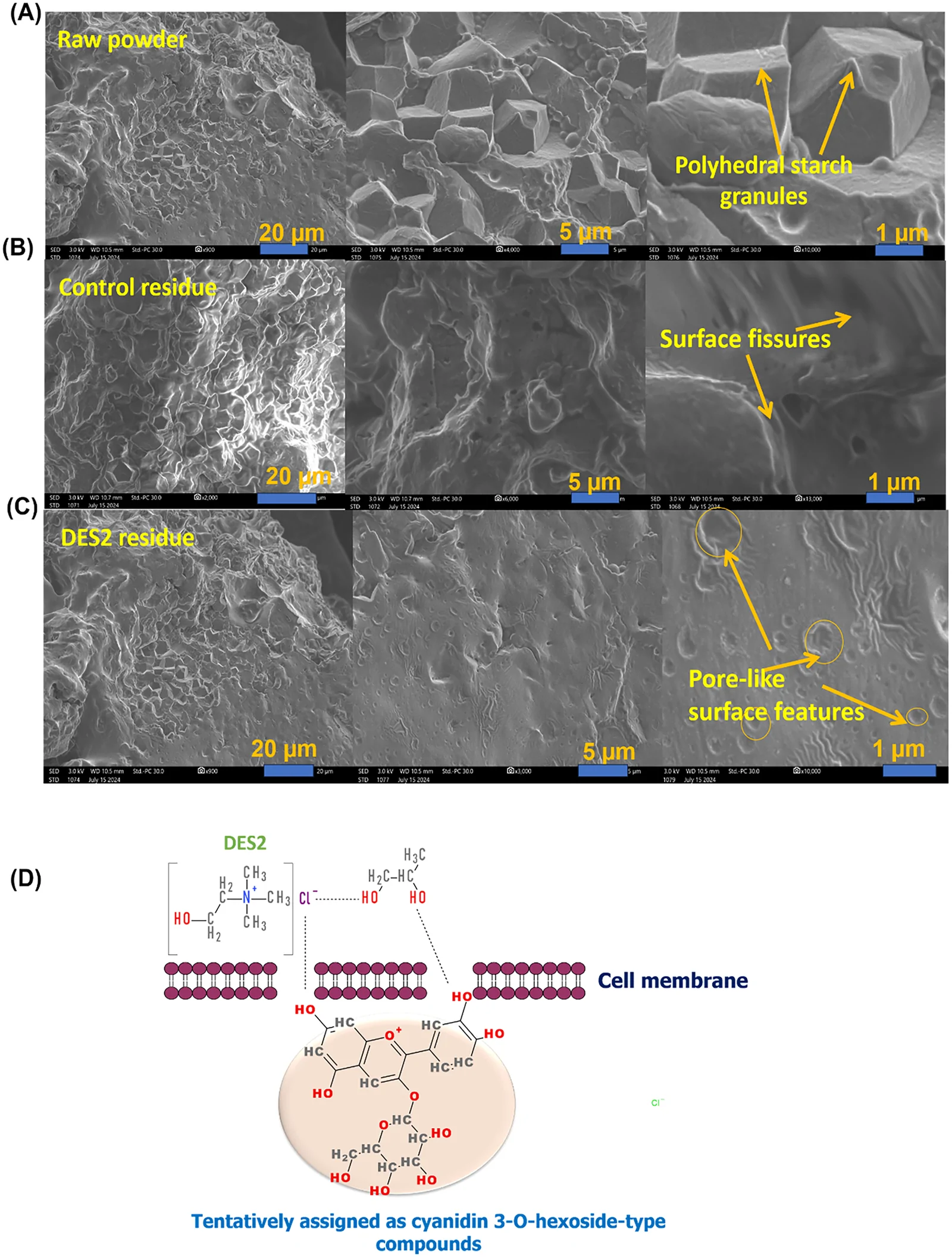
SEM micrographs illustrate the surface morphology of black rice powder before and after extraction: (A) raw powder showing aggregated particles and polyhedral starch granules; (B) control residue after conventional ethanol extraction showing surface erosion and surface fissures; and (C) DES2 residue after ultrasound-assisted extraction showing surface disruption and pore-like surface features. Scale bars from left to right represent 20, 5 and 1 µm, respectively. (D) Proposed working hypothesis for the enhanced recovery of ACN-related compounds tentatively assigned as cyanidin 3-*O*-hexoside-type compounds.

Based on the SEM observations and the chemical composition of the extraction system, a plausible mechanism for the enhanced recovery of ACN-related compounds tentatively assigned as cyanidin 3-*O*-hexoside-type compounds is proposed (figure 4D). Ultrasound-induced cavitation may reduce mass-transfer resistance through disruption of the plant matrix, while hydrogen-bonding interactions between DES2 components and cell-wall constituents may contribute to the release and solubilization of ACN-related compounds. Direct spectroscopic or molecular-dynamics evidence for this mechanism was not obtained in the present study; therefore, the proposed pathway should be regarded as a working hypothesis consistent with previous reports [17,59,60].

**Figure 5.**
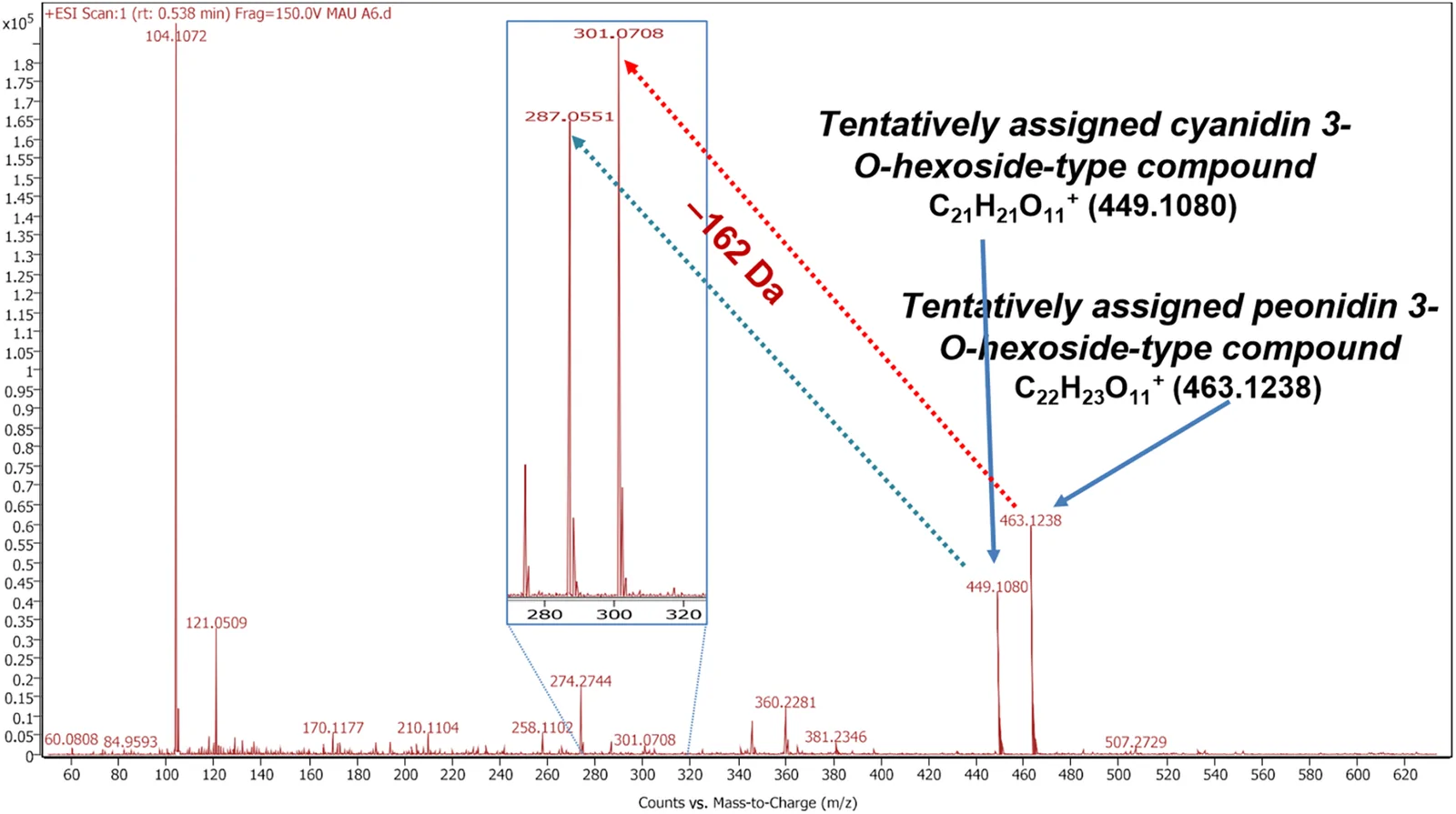
Representative MS/MS spectra showing fragment ions tentatively assigned to ACN-related compounds. The transitions at (*m*/*z*) 463.1238 → 301.0708 and (*m*/*z*) 449.1080 → 287.0551 involve neutral losses of approximately 162 Da, consistent with the loss of a hexose moiety. These assignments are tentative because authentic-standard co-injection was not performed.

The total ACN content of the DES2 extract was quantified using the pH differential method (§2.4.1), independently of chromatographic analysis. Complementary high-resolution QTOF-MS/MS analysis was then used to characterize the major ACN-related signals in the DES2 extract (figure 5). A precursor ion at *m*/*z* 449.1078–449.1092, together with a product ion at *m*/*z* 287.0550–287.0554, was consistent with a cyanidin 3-*O*-hexoside-type compound based on the characteristic loss of 162 Da. Similarly, the precursor ion at *m*/*z* 463.1225–463.1245 and the product ion at *m*/*z* 301.0708–301.0714 were consistent with a peonidin 3-*O*-hexoside-type compound. These compounds were therefore tentatively assigned as cyanidin 3-*O*-hexoside-type and peonidin 3-*O*-hexoside-type ACNs, respectively. The assignments were supported by accurate mass measurements and reported fragmentation patterns, but authentic-standard co-injection was not performed. Consequently, the data do not constitute definitive structural confirmation, and the assigned compounds should be interpreted as putative major ACN constituents of the extract.

### 3.5. Stability of anthocyanins in DES2 extract

#### 3.5.1. Kinetic degradation

Figure 6A,B illustrates the thermal degradation of ACN in the DES2 extract under various storage conditions: 4°C (refrigerator), 25°C (room temperature) and 45°C (incubator). The degradation process was adequately described by a first-order kinetic model under the tested conditions, as evidenced by a strong correlation coefficient and a linear relationship between the logarithm of ACN concentration and time.

**Figure 6.**
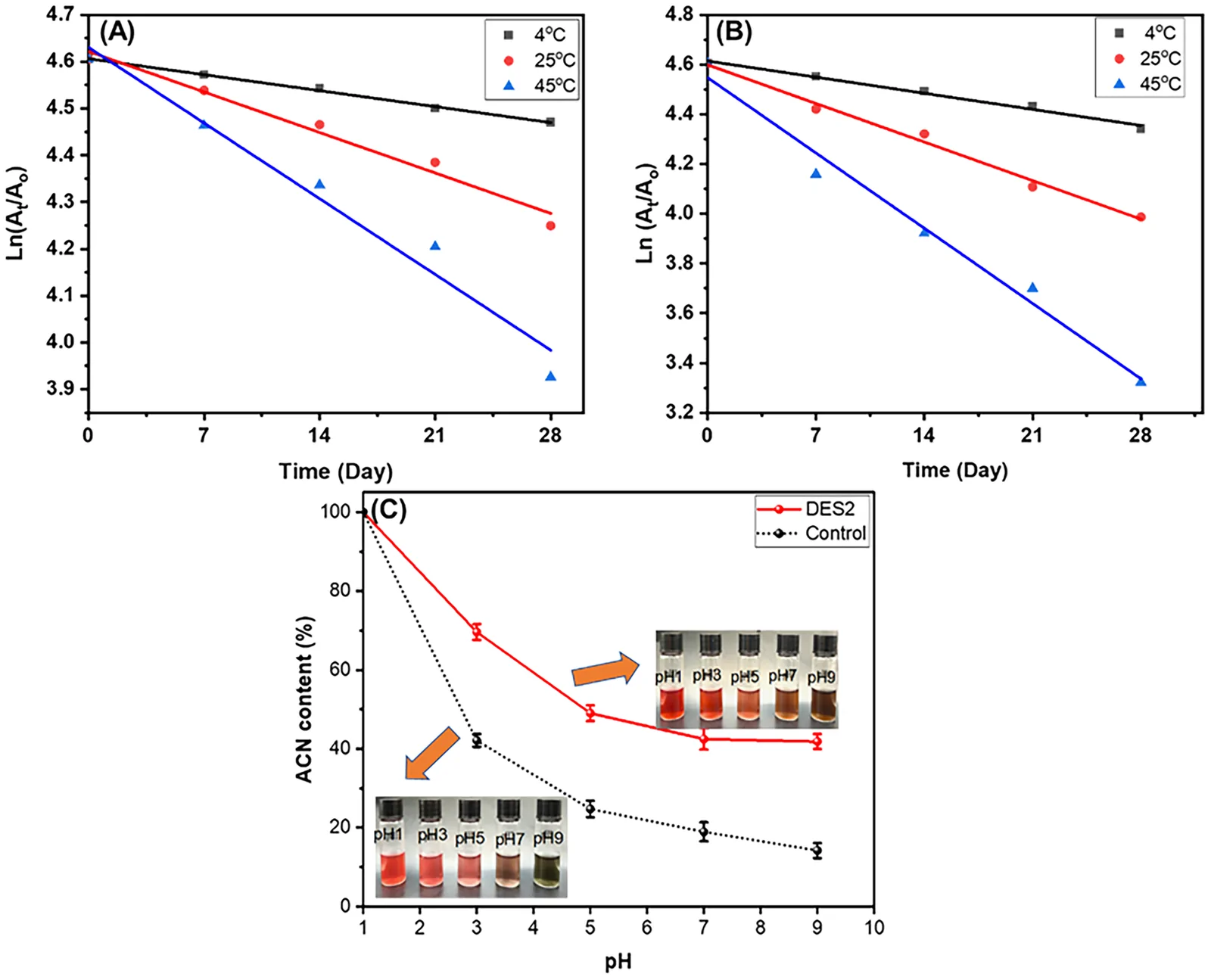
Stability of ACNs in the control extract (A), in DES2 extract (B) and at different pH levels (C).

As the temperature increases, the degradation rate of ACN accelerates. This trend is reflected in the isothermal kinetic parameters calculated using the first-order kinetic model, shown in table 2, which indicate *R*^2^ values ranging from 0.9587 to 0.9963. Notably, at 45°C, the constant degradation rate (*k* = 0.0231 day^−1^) is approximately 4.75 times higher than at 4°C (*k* = 0.00487), while the half-life (*t*_1/2_) decreased from 142.3 days at 4°C to 30.0 days at 45°C, corresponding to an approximately 4.75-fold shorter half-life.

**Table 2.** First-order kinetic parameters for anthocyanin degradation in the DES2 and control extracts at the tested temperatures.

	DES2			control		
treatment (°C)	*k* (day^−^^1^)	*R*^2^	*t*_1/2_ (day)	*k* (day^−^^1^)	*R*^2^	*t*_1/2_ (day)
4	0.00487	0.9963	142.3	0.00926	0.98525	74.9
25	0.01235	0.9639	56.1	0.02213	0.98742	31.3
45	0.0231	0.9587	30.0	0.04321	0.97847	16.0

In control experiments, at 4°C, the rate constant is 0.00926 with a *t*_1/2_ of 74.9 days; at 25°C, *k* is 0.02213 with a *t*_1/2_ of 31.3 days; and at 45°C, *k* is 0.04321 with a *t*_1/2_ of 16.0 days. These findings suggest that ACNs in the DES2 extract showed lower apparent degradation rates than in the control extract under the tested conditions. This result indicates greater retention of the measured ACN content under the tested conditions.

The observed degradation pattern was consistent with previous reports showing a decrease in *t*_1/2_ as the temperature increased, including comparisons between 30 and 45°C [7] and between 60 and 90°C [39]. In the present study, the shorter *t*_1/2_ at higher temperature indicates the thermal sensitivity of ACNs in the black rice extract. Compared with values reported for *Hibiscus sabdariffa* and raspberry juice, the thermal behaviour of the black rice extract appeared different under the respective experimental conditions; however, direct comparisons should be made cautiously because the ACN composition, matrix and storage conditions varied among studies.

ACN degradation may involve hydration of the flavylium cation, ring opening, cleavage of glycosidic bonds, oxidation and subsequent condensation or polymerization reactions [61]. These transformations can alter the chromophore structure and result in reductions in measurable ACN content and colour intensity.

In contrast, the DES2 extract showed relatively greater ACN retention and lower apparent degradation rates than the conventional solvent extract under the storage temperatures tested. This difference may be associated with interactions between ACNs and the components of DES2, including possible hydrogen bonding between hydroxyl groups in the ACNs and propanediol. Such interactions may contribute to the retention of ACNs and the preservation of colour during storage. However, the specific molecular mechanisms underlying this effect were not directly investigated in the present study. DES2 may therefore warrant further investigation as a medium for stabilizing ACN-rich extracts, particularly in food-related formulations.

#### 3.5.2. Thermodynamic analysis

Table 3 presents the calculated results of activation enthalpy (Δ*H*), apparent activation free-energy descriptor (Δ*G*) and entropy (Δ*S*) for the thermal degradation of ACN at each temperature for both DES2 and control samples. The positive apparent Δ*H* values are consistent with a temperature-dependent degradation process under the applied kinetic model with calculated values of 25.27, 25.10 and 24.93 kJ mol^−1^ for DES2 at 277.15, 298.15 and 318.15 K, respectively. In comparison, the control samples show slightly higher Δ*H* values of 25.44, 25.28 and 27.92 kJ mol^−1^ at the same temperatures. This apparent activation-parameter difference should not be interpreted independently of the observed rate constant, because the degradation rate also depends on the pre-exponential factor and the extract matrix. The positive enthalpy values are consistent with the temperature-dependent nature of the degradation process. However, these parameters should be interpreted as apparent kinetic descriptors under the tested model and conditions; they do not, by themselves, establish differences in ACN structural stability or the molecular mechanisms of degradation.

**Table 3.** Thermodynamic parameters for the thermal degradation process of anthocyanins in the extract.

	DES2			control		
*T* (K)	Δ*H* (kJ mol^−1^)	Δ*G* (kJ mol^−1^)	Δ*S* (J (mol K)^−1^)	Δ*H* (kJ mol^−1^)	Δ*G* (kJ mol^−1^)	Δ*S* (J (mol K)^−1^)
277.15	25.27	80.25	−198.37	25.44	78.77	−192.40
298.15	25.10	83.84	−197.04	25.28	82.39	−191.58
318.15	24.93	87.64	−197.10	27.92	85.98	−182.49

As the temperature increases, the degradation rates for both samples follow a similar trend, with the Δ*G* values remaining positive across all temperatures. Specifically, Δ*G* for DES2 increases from 80.25 kJ mol^−1^ at 277.15 K to 87.64 kJ mol^−1^ at 318.15 K. In contrast, the control samples exhibit Δ*G* values of 78.77, 82.39 and 85.98 kJ mol^−1^, respectively. The higher calculated Δ*G* values should be interpreted as differences in the apparent model-derived descriptors, rather than direct evidence of a lower degradation tendency. The apparent activation free energy (Δ*G*) represents the balance between the energy available to do work and the energy exchanged within the system. The positive Δ*G* values suggest that ACN degradation was less thermodynamically favourable under the applied model and experimental conditions. However, these values should be interpreted as apparent, model-derived thermodynamic descriptors rather than as direct evidence that degradation was non-spontaneous or that additional energy input was required.

The Δ*S* values provide additional insight into the reaction dynamics. The values are consistently negative for both samples: −198.37, −197.04 and −197.10 J (mol K)^−1^ for DES2; versus −192.40, −191.58 and −182.49 J (mol K)^−1^ for the control.

The negative Δ*S* values describe the apparent entropy changes calculated from the model; they do not directly establish transition-state ordering. The more negative values observed for the DES2 samples may reflect differences in the transition-state characteristics and apparent degradation behaviour of the two extract systems. Nevertheless, these model-derived parameters should not be interpreted as direct confirmation of molecular ordering, lower intrinsic reactivity or ACN structural stability.

The thermal degradation data showed lower apparent degradation rates and longer half-lives for ACNs in the DES2 extract than in the control samples under the tested storage conditions. The calculated activation parameters also differed between the two systems, including differences in Δ*H*, Δ*G* and Δ*S*. These parameters should be interpreted as apparent, model-derived thermodynamic descriptors rather than direct evidence of ACN structural stability, molecular ordering or degradation spontaneity. Overall, the results suggest that DES2 may contribute to improved ACN retention during storage under the conditions investigated; however, further structural and mechanistic studies are required to determine the basis of this effect.

#### 3.5.3. Effect of pH

The effects of pH adjustment on the ACN content and colour of the extracts are shown in figure 6C and figures S9 and S10 in the supplementary material. At pH 1, both the DES2 extract and the control sample exhibited a red colour, consistent with the predominance of ACNs in the cationic flavylium form, which is generally associated with red to purple hues and an absorption maximum near 520 nm [62]. When the pH increased to 3, the red colour of the DES2 extract was largely maintained, whereas the control extract gradually lost colour, accompanied by a marked decrease in absorbance at 520 nm. This change may reflect a shift in the ACN equilibrium towards less-coloured or colourless forms in the control sample. At pH 5, carbinol pseudo-base and colourless chalcone forms are expected to become more prevalent [63,64]. Nevertheless, the four commonly described structural forms of ACNs may coexist and remain in pH-dependent equilibrium over the pH range of 4–6 [65].

The stability and colour response of ACNs depend on the substitution pattern of the benzene ring and on the presence of hydroxyl and methoxyl groups. Aglycones are generally less stable under near-neutral conditions, whereas mono- and diglycosylated derivatives may show greater stability because sugar residues can influence their reactivity and degradation pathways [66]. The pH-dependent behaviour observed here may therefore be related, at least in part, to the DES2 matrix; however, the molecular basis of this effect was not directly investigated.

At pH 7, the ethanol extract became bluish-purple. The absorbance around 520 nm substantially decreased, while a new band appeared at approximately 620 nm. This spectral change is consistent with a shift towards quinonoid forms or related deprotonated species, which are commonly associated with purple to blue coloration [62]. In contrast, the DES2 extract retained a deep-pink colour. Under alkaline conditions at pH 9, the ethanol extract appeared green, and the absorbance at 620 nm increased markedly. These changes are consistent with further structural transformation and degradation of ACNs at high pH. The response may vary among individual ACNs; for example, cyanidin, delphinidin and pelargonidin derivatives may undergo substantial changes under alkaline conditions, whereas methylated ACNs such as peonidin may display different colour responses [67]. A petunidin glycoside reported in the literature to remain relatively stable at higher pH has also been associated with a blue-purple coloration [62]. Because the individual ACNs responsible for the observed spectral changes were not confirmed using authentic standards, these assignments should be considered interpretive rather than definitive.

Notably, the DES2 extract maintained its pink colour, and the UV–visible spectrum showed minimal changes at pH 5, 7 and 9. The relatively small spectral changes observed for the DES2 extract may reflect matrix effects; the specific molecular basis was not determined.

### 3.6. Nanoliposome loading of the DES2 extract (NLDES2)

#### 3.6.1. NLDES2 characterization

Nanoliposomes loaded with the DES2 extract were prepared using the solvent-injection method. The zeta potential of the system was measured at −27.1 mV. The negative zeta potential indicates a negatively charged vesicle surface and may contribute to electrostatic repulsion among the nanoliposomes; however, zeta potential alone does not establish dispersion stability (figure 7A). The DLS data showed that NLDES2 vesicles had a Z-average diameter of 201.3 nm (figure 7B). A PDI of 0.228 indicated a relatively narrow size distribution under the measurement conditions; a PDI greater than 0.5 is generally considered to indicate a non-uniform size distribution. However, PDI alone does not establish dispersion or storage stability [68]. TEM analysis showed approximately spherical vesicles with a size distribution ranging from 150 to 350 nm and a mean image-based diameter of 185 nm (figure 7C,D). The difference between the DLS-derived Z-average (201.3 nm) and the TEM-based mean size (185 nm) may reflect the different measurement principles, including the hydrated state of the vesicles during DLS analysis and the dried state of the samples during TEM preparation; this difference does not necessarily indicate a discrepancy between the two methods. The EE was 73.8 ± 4.3%, indicating that a substantial proportion of the measured ACN content was associated with the nanoliposomal formulation.

**Figure 7.**
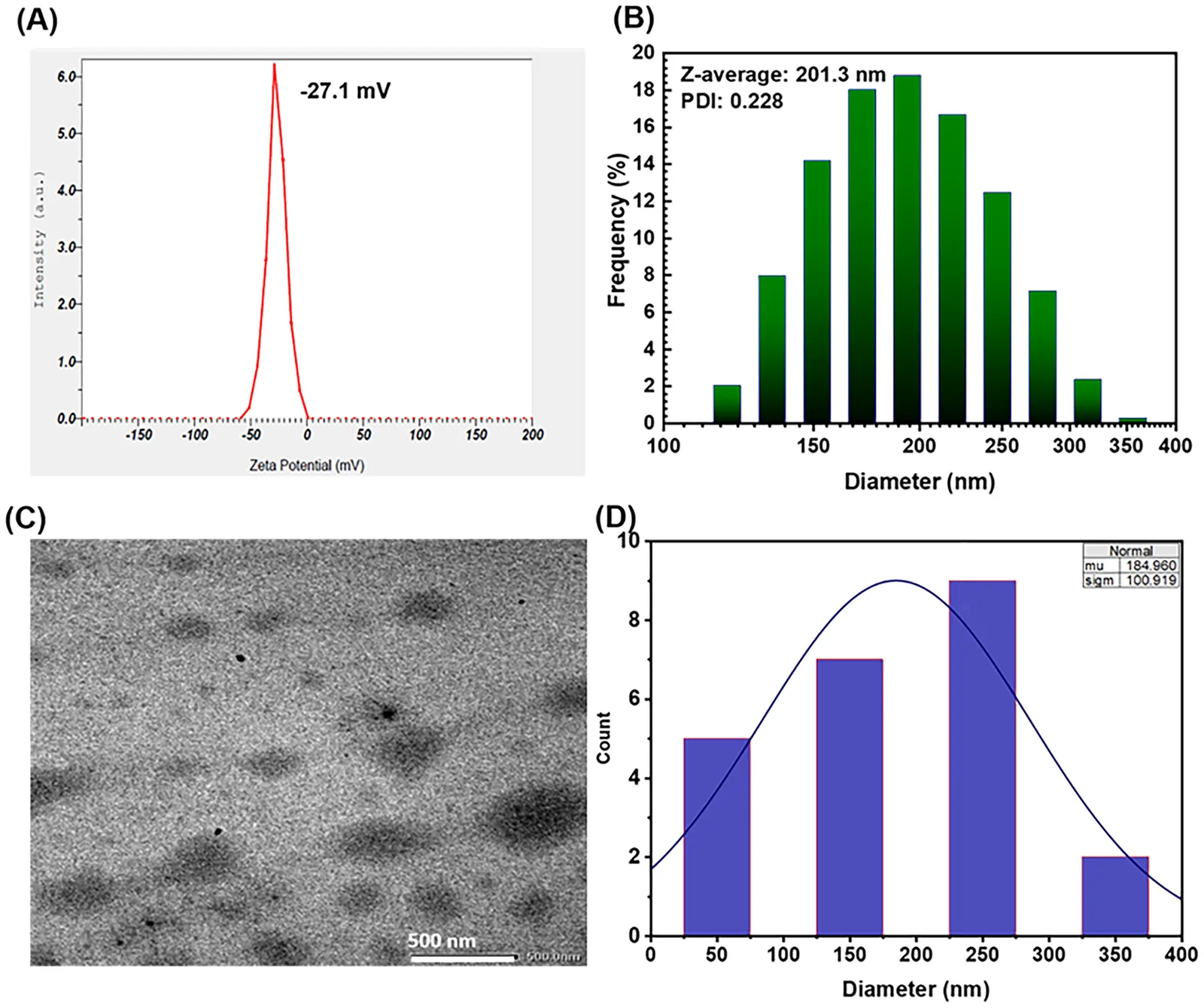
(A) Zeta potential; (B) particle-size distribution measured by DLS; (C) vesicle morphology observed by TEM (scale bar, 500 nm); and (D) ImageJ analysis of TEM images of the NLDES2 sample.

#### 3.6.2. NLDES storage stability

The physico-chemical stability of the ACN-loaded nanoliposomes was evaluated by monitoring changes in particle size, PDI, zeta potential and ACN retention rate at two storage temperatures (4 and 25°C) over 28 days. The results (table 4) demonstrated that storage temperature significantly affected the measured particle size, PDI, zeta potential and ACN retention rate (*p* < 0.05, one-way ANOVA/Tukey's test at each storage time point). Under refrigerated conditions (4°C), only modest changes in particle size, PDI and zeta potential were observed over 28 days. The measured ACN retention was 89.83 ± 0.65% at day 28. At room temperature (25°C), the liposomal dispersion showed poorer storage stability than the formulation stored at 4°C. After 28 days, the mean particle size increased from 201.00 to 345.50 nm, while the PDI increased to 0.535, indicating a broader and more heterogeneous particle-size distribution. The zeta potential also shifted from its initial value to −18.00 mV, accompanied by a decrease in ACN retention to 55.03%. These changes suggest substantial alterations in the colloidal properties of the formulation during storage and may be consistent with particle aggregation and/or vesicle destabilization; however, the underlying mechanisms were not directly determined in this study.

**Table 4.** Storage stability of anthocyanin-loaded nanoliposomes at 4 and 25°C over a 28-day period.

temperature (°C)	time (days)	particle size (nm)	PDI	zeta potential (mV)	ACN retention (%)
4	0	201.00 ± 0.44e	0.228 ± 0.003g	−27.10 ± 0.36e	100.00 ± 0.00a
	7	202.50 ± 0.70e	0.231 ± 0.003fg	−26.87 ± 0.35e	97.93 ± 0.66ab
	14	204.27 ± 0.85e	0.235 ± 0.003fg	−26.47 ± 0.35de	95.17 ± 0.65de
	21	206.57 ± 1.07e	0.242 ± 0.003ef	−26.17 ± 0.35de	92.50 ± 0.70cd
	28	209.17 ± 1.23de	0.249 ± 0.003e;	−25.70 ± 0.36d	89.83 ± 0.65de
25	0	201.00 ± 0.44e	0.228 ± 0.003g	−27.10 ± 0.36e	100.00 ± 0.00a
	7	218.37 ± 3.10d	0.268 ± 0.004d	−25.50 ± 0.36d	88.27 ± 1.06e;
	14	245.50 ± 4.86c	0.329 ± 0.005c	−23.50 ± 0.40c	77.17 ± 1.23f
	21	288.83 ± 6.56b	0.420 ± 0.006b	−21.00 ± 0.40b	66.17 ± 1.46g
	28	345.50 ± 7.16a	0.535 ± 0.007a	−18.00 ± 0.44a	55.03 ± 1.67h

Values are expressed as mean ± standard deviation (*n* = 3). Different lowercase letters within the same column indicate statistically significant differences (*p* < 0.05) according to Tukey's test.

Under the tested conditions, storage at 4°C was therefore more suitable for maintaining the measured physico-chemical properties and ACN retention of the formulation. To improve stability at ambient temperature, future formulation studies could evaluate solid-state approaches, such as lyophilization or spray drying with suitable protective excipients, as well as surface-modification strategies. The effects of these approaches on particle characteristics, ACN retention, reconstitution performance and biological activity should be assessed before their practical or commercial application is considered.

### 3.7. Anti-oxidant and antibacterial evaluation

The bioactivity of the DES2 extract, blank DES2 solvent (DES2-free) and nanoliposomal formulation (NLDES2) was evaluated in terms of anti-oxidant and antibacterial activity (figure 8).

**Figure 8.**
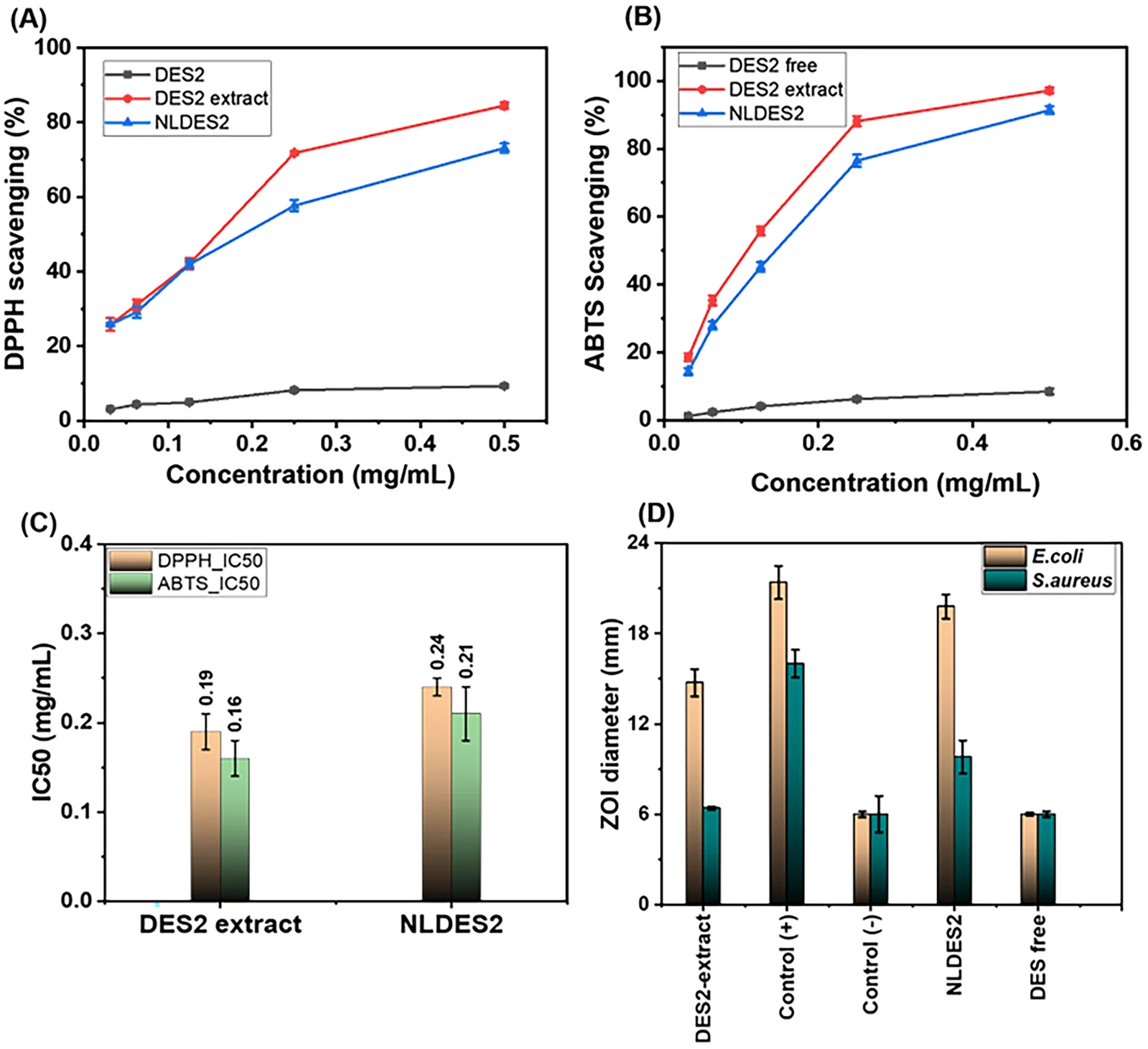
Anti-oxidant and antibacterial evaluation of DES2-free, DES2 extract and NLDES2: (A) DPPH radical-scavenging activity; (B) ABTS radical-scavenging activity; (C) IC50 values (mg ml^−1^) for the DPPH and ABTS assays; and (D) zones of inhibition (ZOI, mm) against selected Gram-positive and Gram-negative bacterial strains. The IC50 value of DES2-free is not presented in (C) because its radical-scavenging activity remained insufficient to reach 50% inhibition within the tested concentration range. Data are expressed as mean ± standard deviation (*n* = 3).

#### 3.7.1. Anti-oxidant activity

The DPPH and ABTS radical-scavenging activities were evaluated over a concentration range of 0.03125–0.5 mg ml^−1^ (figure 8A,B), and the corresponding IC50 values are presented in figure 8C. Both DES2 extract and NLDES2 showed concentration-dependent increases in radical-scavenging activity. In contrast, DES2-free exhibited limited activity, with the maximum scavenging percentage remaining below 10% at the highest tested concentration. Accordingly, its IC50 value was not determined within the tested concentration range and was therefore reported as greater than 0.5 mg ml^−1^.

At 0.5 mg ml^−1^, DES2 extract showed DPPH and ABTS scavenging percentages of 84.49 and 97.20%, respectively. The corresponding IC50 values were 0.19 ± 0.06 mg ml^−1^ for DPPH and 0.16 ± 0.08 mg ml^−1^ for ABTS. NLDES2 showed scavenging percentages of 73.11% for DPPH and 91.40% for ABTS at the same concentration, with IC50 values of 0.24 ± 0.05 and 0.21 ± 0.06 mg ml^−1^, respectively.

These results indicate that DES2 extract reached 50% radical-scavenging activity at a lower concentration than NLDES2 under the tested conditions. The slightly higher IC50 values and lower scavenging percentages observed for NLDES2 may reflect differences in sample accessibility, dispersion, dilution behaviour, matrix effects or interactions between the ACN-rich extract and the lipid and aqueous phases. Encapsulation may influence the accessibility of ACNs to the radicals during the assay. Therefore, the lower activity measured for NLDES2 at the selected incubation time should be interpreted as an assay-dependent difference and should not be taken as evidence of a complete loss of anti-oxidant activity.

The anti-oxidant assays used in this study measured radical-scavenging activity after a fixed incubation time and were not designed to determine ACN release kinetics. Consequently, the present results do not demonstrate controlled, sustained or extended release from the nanoliposomal formulation. They only indicate that NLDES2 retained measurable radical-scavenging activity under the assay conditions. Direct assessment of release behaviour would require dedicated time-resolved *in vitro* release studies measuring ACN transfer from the nanoliposomes into a defined release medium.

For both DES2 extract and NLDES2, the measured scavenging percentages were higher in the ABTS assay than in the DPPH assay. This difference may partly reflect the greater compatibility of the hydrophilic ACN-rich extract with the aqueous reaction medium used in the ABTS assay. However, assay-specific factors may also contribute to the observed difference.

#### 3.7.2. Antibacterial activity

The antibacterial performance of the prepared samples was evaluated against representative Gram-negative (*E. coli*) and Gram-positive (*S. aureus*) strains using the agar well diffusion method, quantified by the zone of inhibition (ZOI, mm) (figure 8D).

Assay validity was assessed using the control groups. The positive control produced inhibition zones of 24.1 ± 1.1 mm against *E. coli* and 16.0 ± 0.9 mm against *S. aureus*. The negative control (Control (−)) and blank solvent (DES2-free) produced no measurable inhibition beyond the well diameter, with a recorded zone of 6.0 mm. These results indicate the absence of detectable antibacterial activity from the vehicle under the assay conditions.

Both DES2 extract and NLDES2 produced measurable inhibition zones against *E. coli* under the agar-well diffusion conditions used in this study, whereas inhibition against *S. aureus* was limited. DES2 extract produced a zone of inhibition of 14.7 ± 0.9 mm against *E. coli*, while NLDES2 produced a larger zone of 19.8 ± 0.8 mm. These results indicate preliminary, assay-dependent antibacterial activity rather than confirmed antibacterial potency. The larger inhibition zone observed for NLDES2 may reflect differences in diffusion, dispersion or sample–agar interactions. The mechanism responsible for the observed inhibition was not determined. All samples showed limited inhibition against *S. aureus* under the conditions investigated. Minimum inhibitory concentration and minimum bactericidal concentration measurements, together with mechanistic studies, would be required for a more rigorous evaluation of antibacterial efficacy.

This difference in inhibition-zone size against *E. coli* may reflect differences in dispersion, diffusion through the agar, or the availability of encapsulated ACNs and other co-extracted phenolic compounds. However, the agar-diffusion assay does not establish a specific antibacterial mechanism; therefore, effects, such as membrane disruption, intracellular interference, cell-wall penetration or liposome–membrane fusion, should be regarded as possible explanations requiring further investigation. In addition, the ZOI values should be interpreted cautiously because the tested formulations were not necessarily normalized to the same ACN concentration.

Against *S. aureus*, DES2 extract and NLDES2 produced ZOIs of 6.4 ± 0.1 and 9.8 ± 1.1 mm, respectively. Because the baseline well diameter was 6.0 mm, the DES2 extract value represented little or no inhibition beyond the well, whereas the larger NLDES2 zone indicated limited inhibition under the tested conditions. These results suggest lower apparent activity against *S. aureus* than against *E. coli*. The difference may be related to species-specific susceptibility and/or differences in sample diffusion; however, the contribution of the Gram-positive cell envelope cannot be confirmed from the present assay alone. Additional tests, such as minimum inhibitory concentration, minimum bactericidal concentration, time-kill analysis or membrane-integrity assays, would be required to establish the underlying mechanism.

## 4. Conclusion

This study evaluated a DES-assisted ultrasound extraction process and a subsequent nanoliposomal formulation for ACN-rich extracts from black rice (*O. sativa* L.). Among the DES systems examined, choline chloride/1,2-propanediol (DES2) produced the highest ACN content under the optimized extraction conditions, corresponding to a 3.33-fold increase relative to the conventional ethanol-based method. High-resolution QTOF-MS/MS analysis supported the tentative assignment of cyanidin 3-*O*-hexoside-type and peonidin 3-*O*-hexoside-type compounds as major constituents of the DES2 extract. Because authentic reference standards were not used, these assignments should not be considered definitive structural identifications.

The DES2 extract exhibited lower apparent degradation rates and longer half-life than the control under the investigated thermal conditions at pH 1, with a substantially longer half-life at 45°C. These results indicate improved retention of the measured ACN content in the DES2 extract under the conditions tested. The calculated activation parameters (Δ*H*, Δ*G* and Δ*S*) are apparent, model-derived descriptors obtained from the degradation kinetics and should therefore be interpreted cautiously. They do not, on their own, provide definitive evidence of molecular stabilization, transition-state organization, intrinsic ACN reactivity or the spontaneity of the degradation process.

The DES2 extract showed anti-oxidant activity in the assays performed and produced measurable inhibition zones against the tested bacterial strains. NLDES2 generated a larger inhibition zone against *E. coli* than the free DES2 extract under the agar-diffusion conditions used in this study. However, agar-diffusion results are influenced by factors such as sample diffusion, dispersion and compound availability; therefore, the observed difference should not be interpreted as definitive evidence of enhanced antibacterial delivery or a specific antibacterial mechanism.

NLDES2 showed an EE of 73.8 ± 4.3% and retained 89.83 ± 0.65% of the measured ACN content after 28 days at 4°C. These findings indicate that NLDES2 can retain a substantial proportion of the extract's measured ACN content under refrigerated conditions. Nevertheless, the present data are insufficient to establish long-term physical stability, protection against all degradation pathways, enhanced bioavailability or biological efficacy.

Overall, the results support DES2-assisted extraction and nanoliposomal formulation as promising approaches for recovering and formulating ACN-rich black rice extracts. However, the sustainability of the complete workflow and its suitability for food, nutraceutical, cosmetic or pharmaceutical applications require separate evaluation and cannot be established solely from the present findings. Future work should include confirmation with authentic standards, comprehensive quantitative ACN profiling, complementary anti-oxidant assays, such as FRAP and ORAC, and quantitative antimicrobial analyses, including minimum inhibitory concentration, minimum bactericidal concentration and time-kill assays. Simulated gastrointestinal release studies, release-mechanism modelling and *in vivo* investigations are also needed to clarify the performance and potential applications of NLDES2.

## Supplementary Material



## Data Availability

The authors confirm that all data supporting the findings of this study are available within the article and its supplementary material [69]. The supplementary material contains several supplementary figures and tables referenced in the main text, providing additional experimental details, characterization data and raw spectra for the synthesis and application of choline chloride-based DESs. Specifically, FTIR spectra of various DES compositions (supplementary material, figures S1–S7), visual evidence of extraction-related colour changes (supplementary material, figure S8) and pH-dependent absorption spectra (supplementary material, figures S9 and S10) are also provided to support the discussion in the main text.
