# Supplementary material for: Choline chloride-based deep eutectic solvents for efficient anthocyanin recovery from black rice: process, kinetics and nanoliposomal formulation
Source: R Soc Open Sci. 2026 Oct 14;13(10):260927. doi: 10.1098/rsos.260927 (PMC13682423; doi:10.1098/rsos.260927)
Supplement: Supplementary file 1 [file rsos.260927.zip › rsos.260927/rsos260927_si_001.docx]

THE ELECTRONIC SUPPORTING INFORMATION

**Choline Chloride-based Deep Eutectic Solvents for Efficient Anthocyanins Recovery from Black Rice: Process, Kinetics, and Nanoliposomal Formulation**

**Tran Quang Hieu^1*^, Nguyen Ba Thanh^2^, Nguyen Quynh Dao^3^, Pham Quoc Dat^3^, Nguyen Thi Ngoc Tram^3^, Nguyen Thanh Danh^4^, Nguyen Quoc Thang^1^, Le Van Tan^1^**

^1^Faculty of Chemical Engineering, Industrial University of Ho Chi Minh City, Ho Chi Minh City 700000, Viet Nam, 12 Nguyen Van Bao, Hanh Thong Ward, Ho Chi Minh City 700000, Viet Nam.

^2^Institute Biotechnology and Food Technology, Industrial University of Ho Chi Minh City, 12 Nguyen Van Bao, Hanh Thong Ward, Ho Chi Minh City 700000, Viet Nam.

^3^Faculty of Food Technology, Saigon Technology University, 180 Cao Lo, Chanh Hung Ward, Ho Chi Minh City 700000, Viet Nam.

*^4^Institute of Advanced Technology, Vietnam Academy of Science and Technology, 01B, TL29, Thanh Loc Ward, Ho Chi Minh City, 700000 Viet Nam.*

^*^Corresponding author, email address: [*tranquanghieu@iuh.edu.vn*](mailto:tranquanghieu@iuh.edu.vn)

This file contains the Electronic Supplementary Information (ESI) supporting the research article published in Royal Society Open Science. The provided data includes detailed experimental parameters and spectroscopic characterizations essential for the extraction and analysis of anthocyanins from black rice using green Deep Eutectic Solvents (DES).

**TABLE OF CONTENTS**

Figure S1. IR of DES1

Figure S2. IR of DES2

Figure S3. IR of DES3

Figure S4. IR of DES4

Figure S5. IR of DES5

Figure S6. IR of DES6

Figure S7. IR of DES7

Figure S8. **Sample images of black rice flour before and after extraction; (A): rice flour, (B) ethanol extract residue, (C) DES2 extract residue**

Figure S9. Absorption spectra of Ethanol-H_2_O extracts with different pH.

Figure S10. Absorption spectra of DES2- extracts with different pH.

Figure S1. IR of DES1

Figure S2. IR of DES2

Figure S3. IR of DES3

Figure S4. IR of DES4

Figure S5. IR of DES5

Figure S6. IR of DES6

Figure S7. IR of DES7

Figure S8. **Sample images of black rice flour before and after extraction; (A): rice flour, (B) ethanol extract residue, (C) DES2 extract residue**

Figure S9. Absorption spectra of Ethanol-H_2_O extracts with different pH.

Figure S10. Absorption spectral of DES2- extracts with difference PH.
